# Treatment of Chronic Large and Persistent Macular Hole by a new technique in a Tertiary Care Hospital

**DOI:** 10.12669/pjms.37.4.3618

**Published:** 2021

**Authors:** Tanweer Hasan Khan, Syed Fawad Rizvi, Syed Asaad Mahmood, Lubna Feroz

**Affiliations:** 1 Dr. Tanweer Hasan Khan, FCPS (Ophth). Consultant Ophthalmologist, LRBT Tertiary Eye Care Hospital, Korangi 2 ½, Karachi, Pakistan; 2 Dr. Syed Fawad Rizvi, FCPS (Ophth). Chief Consultant Ophthalmologist, LRBT Tertiary Eye Care Hospital, Korangi 2 ½, Karachi, Pakistan; 3 Dr. Syed Asaad Mahmood, FCPS (Ophth), Ophthalmologist, LRBT Tertiary Eye Care Hospital, Korangi 2 ½, Karachi, Pakistan; 4 Dr. Lubna Feroz, FCPS (Ophth). Ophthalmologist, LRBT Tertiary Eye Care Hospital, Korangi 2 ½, Karachi, Pakistan

**Keywords:** Macular hole, Hydrodissection, Vitrectomy

## Abstract

**Objective:**

To assess the anatomical and functional outcomes of treating chronic persistent large macular hole by macular hole hydrodissection technique in a tertiary eye care hospital.

**Methods:**

This interventional case series study was conducted in the Vitreoretinal department of LRBT Tertiary Teaching Eye Hospital, Karachi, from October 2017 to March 2018, with follow-ups till February 2019. The study included eighteen cases of chronic (symptoms of loss of central vision ≥ 2years), persistent (previously failed macular hole surgery), large (aperture diameter of ≥ 400µm) macular hole. Out of the eighteen patients, eight (44.4%) were males and ten (55.6%) were females. All operated patients underwent macular hole hydrodissection by balanced salt solution using a silicone soft tip extrusion cannula. Patients were followed up post operatively to assess post-operative complications and surgical results.

**Results:**

Among eighteen patients with a mean aperture diameter of 477.1±102.9 µm and basal diameter of 849.4± 92.6µm, complete anatomical closure was achieved in sixteen (88.8%). Five (27.7%) out of the eighteen patients achieved best corrected visual acuity improvement of 6/36, whereas seven (38.8%) patients reached up to a BCVA of 6/60, with maximum improvement of two lines. The mean post-operative follow-up was 332.3± 46.7 days.

**Conclusion:**

Macular hole hydrodissection is a relatively new emerging technique with promising results for the closure of chronic persistent large macular hole.

## INTRODUCTION

Macular hole is one of the chief vitreoretinal disorders which cause metamorphopsia and deprived central vision in the elderly.^1^ The overall incidence is approximately 3.3% per 1000.^2^ The pathogenesis of idiopathic macular hole has been ascribed to the presence of tangential and anteroposterior traction on the fovea by pre foveal cortical vitreous.^3^ It has been postulated that the principal factors for spontaneous closure of macular hole are release of vitreofoveal traction or glial proliferation.^4^

Although vitreous surgery was first described as a probable treatment for Full-Thickness Macular Holes (FTMHs), it has become a customary surgical procedure. With modern surgical techniques, approximately 90% of FTMHs attain anatomical closure on primary surgery, with almost half of patients achieving visual acuity of 20/50 or better.^5^ The closure rate and visual outcome of FTMHs depend on its size and chronicity. Therefore, large chronic FTMHs are known to have a less constructive outcome. Furthermore, FTMHs that failed to close after primary surgery have a poorer prognosis for successful closure after reoperation.^5^ Literature has reported a closure rate of 33% to 80% and 56% for chronic and large macular holes.^6^,^7^ The rate of closure is further compromised in the mutual presence of risk factors including large aperture diameter, chronicity and previously failed surgery for multipart cases of macular hole.^8^

In this study we assessed the anatomical and functional outcomes with the use of a new emerging technique of macular hydrodissection with balance salt solution for the treatment of persistent, chronic, large macular hole of more than 400 µm size.

## METHODS

Prior approval was taken from the hospital ethics board before the start of this study (Ref# LRBT/TTEH/ERC/2531/01, Dated 27/10/2017). This study was conducted in the Vitreoretinal Department of Layton Rehmatulla Benevolent Trust (LRBT) Tertiary Teaching Eye Hospital, Karachi from October 2017 to March 2018, with follow-ups till February 2019; and included eighteen patients of both genders with age ranging from 60 to 80 years. Out of the eighteen patients, eight (44.4%) were males and ten (55.6%) were females. Inclusion criteria comprised of patients having chronic (symptoms of loss of central vision ≥ 2years), persistent (previously failed surgery), large (aperture diameter of > 400µm) macular hole. Patients having no primary surgery or having any other retinal pathology, stage one and two macular hole, less than one year macular hole and traumatic hole were excluded from the surgery. Performa was used to record demographics, brief history, and ocular examination which were done with slit lamp biomicroscopy along with 90D lens. All patients were informed about the study and written consent was obtained individually from each patient. Study approval was obtained from the Ethical review committee and all patients were followed up for a period of one year to assess post-operative complications and surgical results. Data was analyzed through SPSS version 25. Chi squared test was used for comparing pre- and post-operative vision and a p-value of < 0.05 was considered to be significant.

All surgeries were performed under local peri-bulbar anesthesia with a mixture of lidocaine (2%) and bupivacaine (0.7%). A total of 2–3 ml was injected. Under strict aseptic measures, micro-incision vitrectomy using 25 gauge vitrectomy system (Constellation Vision System, Alcon® surgicals) was done. Surgical induction of posterior vitreous detachment (PVD) was done and/or Internal Limiting Membrane (ILM) peeling assisted by brilliant blue (BBG) staining was performed in cases where this had not been done previously or was incomplete. Using a silicone soft tip extrusion cannula, under adequate reflux mode, balanced salt solution was actively refluxed in to the macular hole, lysing the adhesion between the edges of macular hole and retinal pigment epithelium. Thus, basically hydrodissecting the adhesion between macular hole edges and its adjacent retinal pigment epithelium, so as to make the edges of macular hole more mobile for approximation. Then the same soft tip cannula was used to brush the mobile edges closer together. After air-fluid exchange, 20% Sulfur hexafluoride (SF6) gas was injected for internal tamponade. Following surgery, the patients were instructed to strictly observe head down posture for two weeks. OCT macula was done at four to six weeks.

## RESULTS

Eighteen patients of age ranging between 60 to 80 years (mean = 69.4 ± 5.54 years) with chronic, persistent, large macular hole aperture diameter of 477.6 ± 103.11 µm and basal diameter of 849.0± 92.37µm were operated with macular hydrodissection technique during vitrectomy. Complete anatomical closure (defined as flattening and reattachment of the edge of the hole along the entire circumference of the macular hole) was obtained in sixteen patients (88.8%). Five (27.7%) out of the eighteen patients achieved visual acuity improvement of 6/36, whereas seven (38.8%) patients visual acuity reached up to 6/60, with maximum improvement of two lines (p-value = 0.021). The vision remained the same in remaining six (33.3%) patients. The mean post-operative follow-up was 332.3 ± 44.2 days.

## DISCUSSION

Despite the high success rate of modern macular hole surgery, large, recurrent or persistent macular holes remain a challenge for retinal surgeons.^9^ The aim of using this novel surgical technique was anatomical closure and visual improvement in patients who had macular holes which were refractory to all previous treatment.

It is assumed that confiscation of the vitreous and internal limiting membrane (ILM) releases the anteroposterior and tangential traction that leads to the development of the macular hole and allows re-approximation of the hole edges. Furthermore, in light of the hypothesis that macular holes are closed by glial cell proliferation, it has been also proposed that a surgeon can perform ILM peeling to induce a relatively consistent level of trauma to facilitate this gliosis over a FTMH. An added benefit is that the removal of ILM also reduces the possibility of epiretinal membrane formation.^5^

The theory behind the projected technique is that in cases of persistent holes, there is another force at play that impedes approximation of the hole edges, even after reprieve of the abnormal anteroposterior and tangential vitreous traction. This could be caused by the underlying retina, which is too inflexible to permit for edge closure. An important condition for macular holes closing is mobility of the retina around holes.^5^

Several researchers have also reported that macular holes with surrounding subretinal fluid were more likely to close.^10^,^11^ Ideal anatomical and functional outcomes are achieved with a surgical technique that accomplishes the greatest extent of mobilization of macular hole edges, allowing them to re-approximate while causing minimal trauma to the retinal pigment epithelium during mechanical manipulation of the retina.^12^ This can easily be attained if the retina in the macular area was to be released from the underlying retinal pigment epithelium by creating a posterior retinal detachment through sub retinal fluid infusion.^13^

This study achieved anatomical macular hole closure in 88% of cases of chronic large macular hole with the technique of macular hydrodissection. In a similar study but using a different technique for hydrodissecting the macula, Fotis et al achieved 100% anatomical closure in their study.^5^ In another study, Ruban et al. reported 85% anatomical closure when they adopted this technique for traumatic macular hole repair.^14^ Felfili et al reported anatomical closure in 87% of cases.^12^ Despite successful anatomical closure, visual acuity also improved in 66% of cases whereas Oliver et al. reported no subjective change in visual acuity in their study.^15^ Fotis reported visual acuity improvement in 90% of cases.^5^

None of the patient in this study experienced deterioration of pre-existing vision which was similar to the study conducted by Wong R et al.^16^ There were no significant or long-term intra-operative or post-operative complications observed in this study. Sgiziato et al also mentioned no complicationas in their study.^17^ Since this technique involves massaging of the hydrodissected retina with the soft silicone brush, it is expected to be somewhat traumatic to the neurosensory tissue however no impact of this was seen during the follow up period. In a related study, the researchers used the diamond dusted scrapper for massaging the retina after hydrodissecting the macular hole without any long-term visual impairment.^18^ A meta-analysis studying the effects of vitrectomy for idiopathic macular hole reports cataract formation and retinal detachment as the most common complications associated with this procedure.^19^ No such complication was seen during the follow up period in this study.

Overall, macular hole surgery results in increased quality of life even in patients where the fellow eye has good visual acuity.^20^ The technique presented in this study improves the chances of macular hole closure in patients with large macular holes, which otherwise have lower probability of hole closure.^21^ Although this study has shown promising results, it is limited in terms of number of patients and the follow up duration. A larger group of operated eyes and a longer follow up will be required to assess the long-term effects of this procedure.

## CONCLUSION

Micro incision vitreoretinal surgery with dye assisted ILM peeling followed by macular hole hydrodissection showed satisfactory anatomical and visual outcomes in cases of chronic, persistent large macular holes, with previously failed closure obtained after surgery.

### Authors’ Contribution:

**THK and SAM:** conceptualized this study. **SFR and LF:** reviewed the existing relevant clinical studies and finalized the study design. Patient recruitment, data entry at each visit and compilation of records was done by **SAM, LF and THK**. All surgeries were performed by **SFR**. Statistical analysis was performed by **SAM and THK**. Manuscript was prepared by **THK**, edited and corrected by **SAM and LF** and proofread and finalized by **SFR.**

**THK** is responsible and accountable for the accuracy of the work.

## References

[1] Jackson TL, Donachie PHJ, Sparrow JM, Johnston RL (2013). United Kingdom National Ophthalmology Database study of vitreoretinal surgery:report 2, macular hole. Ophthalmology.

[2] Fine SL (1993). Discussion. Ophthalmology.

[3] Khaqan HA, Lubna Jameel F, Muhammad (2016). Visual outcomes of macular hole surgery. J Coll Physicians Surg Pak.

[4] Lo WR, Hubbard GB (2006). Macular hole formation, spontaneous closure, and recurrence in a previously vitrectomized eye. Am J Ophthalmol.

[5] Fotis K, Alexander P, Sax J, Reddie I, Kang CY, Chandra A (2019). Macular Detachment for the Treatment of Persistent Full-Thickness Macular Holes. Retina.

[6] Shukla SY, Afshar AR, Kiernan DF, Hariprasad SM (2014). Outcomes of chronic macular hole surgical repair. Indian J Ophthalmol.

[7] Ip MS, Baker BJ, Duker JS, Reichel E, Baumal CR, Gangnon R (2002). Anatomical outcomes of surgery for idiopathic macular hole as determined by optical coherence tomography. Arch Ophthalmol.

[8] Chow DR, Chaudhary KM (2015). Optical coherence tomography-based positioning regimen for macular hole surgery. Retina.

[9] Tam ALC, Yan P, Gan NY, Lam WC (2018). The current surgical management of large, recurrent, or persistent macular holes. Retina.

[10] Rishi P, Reddy S, Rishi E (2014). Repeat gas insufflation for successful closure of idiopathic macular hole following failed primary surgery. Indian J Ophthalmol.

[11] Hillenkamp J, Kraus J, Framme C, Jackson TL, Roider J, Gabel VP (2007). Retreatment of full-thickness macular hole:Predictive value of optical coherence tomography. Br J Ophthalmol.

[12] Felfeli T, Mandelcorn ED (2019). Macular Hole Hydrodissection:Surgical Technique for the Treatment of Persistent, Chronic, and Large Macular Holes. Retina.

[13] Solomon SD, Dong LM, Haller JA, Gilson MM, Hawkins BS, Bressler NM (2009). Risk factors for rhegmatogenous retinal detachment in the submacular surgery trials:SST report No.22. Retina.

[14] Ruban A, Lytvynchuk L, Zolnikova A, Richard G (2019). Efficiency of the Hydraulic Centripetal Macular Displacement Technique in the Treatment of Traumatic Full-Thickness Macular Holes. Retina.

[15] Oliver A, Wojcik EJ (2011). Macular detachment for treatment of persistent macular hole. Ophthalmic Surg Lasers Imaging.

[16] Wong R, Howard C, Orobona GD (2018). Retina Expansion Technique for Macular Hole Apposition Report 2:Efficacy, Closure Rate, and Risks of a Macular Detachment Technique to Close Large Full-Thickness Macular Holes. Retina.

[17] Szigiato AA, Gilani F, Walsh MK, Mandelcorn ED, Muni RH (2016). Induction of macular detachment for the treatment of persistent or recurrent idiopathic macular holes. Retina.

[18] Mohammed OA, Pai A (2017). New Surgical Technique for Management of Recurrent Macular Hole. Middle East Afr J Ophthalmol.

[19] Parravano M, Giansanti F, Eandi CM, Yap YC, Rizzo S, Virgili G (2015). Vitrectomy for idiopathic macular hole. Cochrane Database Syst Rev.

[20] Hirneiss C, Neubauer AS, Gass CA, Reiniger IW, Priglinger SG, Kampik A (2007). Visual quality of life after macular hole surgery:outcome and predictive factors. Br J Ophthalmol.

[21] Ezra E (2001). Idiopathic full thickness macular hole:natural history and pathogenesis. Br J Ophthalmol.

